# Alteration of the Antioxidant Capacity and Gut Microbiota under High Levels of Molybdenum and Green Tea Polyphenols in Laying Hens

**DOI:** 10.3390/antiox8100503

**Published:** 2019-10-22

**Authors:** Jianping Wang, Zengqiao Yang, Pietro Celi, Lei Yan, Xuemei Ding, Shiping Bai, Qiufeng Zeng, Xiangbing Mao, Bing Feng, Shengyu Xu, Keying Zhang

**Affiliations:** 1 Animal Nutrition Institute, Key Laboratory of Animal Disease-Resistance Nutrition, Ministry of Education, Ministry of Agriculture and Rural Affairs, Sichuan Agricultural University, Chengdu 611130, China; yangzq1994@sina.com (Z.Y.); dingxuemei0306@163.com (X.D.); shipingbai@sicau.edu.cn (S.B.); zqf@sicau.edu.cn (Q.Z.); 13856@sicau.edu.cn (X.M.); fengbin@sicau.edu.cn (B.F.); shengyu_x@hotmail.com (S.X.); 2 Faculty of Veterinary and Agricultural Sciences, The University of Melbourne, Parkville 3010, Australia; Pietro.Celi@dsm.com; 3 DSM Nutritional Products, Wurmisweg 576, 4303 Kaiseraugst, Switzerland; nickyx666@hotmail.com

**Keywords:** antioxidant capacity, gut microbiota, layers, molybdenum, tea polyphenols

## Abstract

High dietary levels of molybdenum (MO) can negatively affect productive performances and health status of laying hens, while tea polyphenol (TP) can mitigate the negative impact of high MO exposure. However, our understanding of the changes induced by TP on MO challenged layers performances and oxidative status, and on the microbiota, remains limited. The aim of the present study was to better understand host (performances and redox balance) and microbiota responses in MO-challenged layers with dietary TP. In this study, 200 Lohmann laying hens (65-week-old) were randomly allocated in a 2 × 2 factorial design to receive a diet with or without MO (0 or 100 mg/kg), and supplemented with either 0 or 600 mg/kg TP. The results indicate that 100 mg/kg MO decreased egg production (*p* = 0.03), while dietary TP increased egg production in MO challenged layers (*p* < 0.01). Egg yolk color was decreased by high MO (*p* < 0.01), while dietary TP had no effect on yolk color (*p* > 0.05). Serum alanine transaminase (ALT), aspartate aminotransferase (AST), and malonaldehyde (MDA) concentration were increased by high MO, while total antioxidant capacity (T-AOC), xanthine oxidase (XOD) activity, glutathione s-transferase (GSH-ST), and glutathione concentration in serum were decreased (*p* < 0.05). Dietary TP was able to reverse the increasing effect of MO on ALT and AST (*p* < 0.05). High MO resulted in higher MO levels in serum, liver, kidney, and egg, but it decreased Cu and Se content in serum, liver, and egg (*p* < 0.05). The Fe concentration in liver, kidney, and eggs was significantly lower in MO supplementation groups (*p* < 0.05). High MO levels in the diet led to lower *Firmicutes* and higher *Proteobacteria* abundance, whereas dietary TP alone and/or in high MO treatment increased the *Firmicutes* abundance and the *Firmicutes/Bacteroidetes* ratio at phylum level. High MO increased the abundance of *Proteobacteria* (phylum), *Deltaproteobacteria* (class), *Mytococcales* (order), and *Nanocystaceae* (family), whereas dietary TP promoted the enrichment of *Lactobacillus agilis* (species). Dietary TP also enhanced the enrichment of *Bacilli* (class), *Lactobacillates* (order), *Lactobacillus* (family), and *Lactobacillus gasseri* (species). Microbiota analysis revealed differentially enriched microbial compositions in the cecum caused by MO and TP, which might be responsible for the protective effect of dietary TP during a MO challenge.

## 1. Introduction

Molybdenum (MO) is an essential trace element for animals and universally distributed in the environment [1] and a critical component of metalloenzymes in organisms [2]. However, MO (45 mg/kg BW) can also have negative effects as demonstrated in several studies where rumen microbial metabolism was altered. Moreover, MO (100 mg/kg diet) can interfere with the bioavailability of other trace elements, such as iron (Fe) and zinc (Zn), especially copper (Cu) leading to Cu deficiency [3,4,5,6]. Other adverse effects that have been observed in animals exposed to high dietary levels of MO include poor growth, achromotrichia, severe diarrhea, and anemia [7]. Previous studies documented that high MO can exacerbate lipid peroxidation, decrease the activity of several antioxidant enzymes, and thus disrupt redox balance [8]. The expression of cell apoptosis-related genes is also altered, resulting in cell injuries and apoptosis [9,10]. Due to its extensive industrial and agricultural utilization, MO environmental concentrations are rapidly increasing raising concerns for its potentially toxic effect when present in high levels in animal feed. However, the effect of high levels of MO in layers is not elucidated yet.

Tea polyphenol (TP) is a natural antioxidant of typical flavonoids and exhibits antioxidant activity by indirectly modulating transcriptional factors and their downstream enzyme activities related redox balance system [11,12,13,14]. Numerous studies demonstrated that feeding laying hens a diet supplemented with 500–600 mg/kg of green tea extract improved egg production, feed efficiency [15,16], egg white quality, and the antioxidant activity of eggs during the late laying period [16,17]. Furthermore, recent studies have indicated that dietary polyphenol-rich sources such as epigallocatechin gallate (EGCG) can modulate the intestinal microbiota, promoting the proliferation of beneficial bacteria and increasing the degree of biodiversity in the gut [18,19,20,21]. However, the majority of TP or EGCG studies have focused on the modulation of lipid metabolism and antioxidant function in humans or mice models, whereas information on the detoxification effect of MO is limited.

Therefore, the objective of this experiment was to evaluate the effect of dietary TP on redox balance, production performances, and changes in cecum microbiota in laying hens exposed to high MO levels. Our hypothesis was that dietary TP would reverse the effect of high MO.

## 2. Materials and Methods

### 2.1. Ethics Statement

This study was approved by the guidelines (SYXK2014-187) of the Animal Care and Use Committee of Sichuan Agricultural University and meets the guidelines set by the Regulations for the Administration of Affairs Concerning Experimental Animals of the State Council of the People’s Republic of China. 

### 2.2. Preparation of TP

The TP was purchased from Red Star Pharmaceutical (Co. Ltd. Anhui, China), with 98.6% purity, which contains epigallocatechin gallate (EGCG) of 66.3%, epigallocatechin (EGC) of 16.5%, epicatechin-3-gallate (ECG) of 7.8%, epicatechin (EC) of 5.7%, and caffeine of 0.4%.

### 2.3. Birds, Experimental Design, and Sample Collection

A total of 200 old Lohmann laying hens (65-week-old) were kept in cages (10 hens/cage) and randomly allocated to the following dietary treatments in a 2 × 2 factorial design (2 doses of MO × 2 doses of TP): (1) CON = MO (0 mg/kg) + TP (0 mg/kg); (2) MO = MO (100 mg/kg) + TP (0 mg/kg); (3) TP = MO (0 mg/kg) + TP (600 mg/kg); (4) MT = MO (100 mg/kg) + TP (600 mg/kg). The dose of TP chosen for this study is in line with that used in previous in vivo studies [16,17]. Each treatment consisted of 5 replicates with 10 hens each; layers received the dietary treatments for 12 weeks. The basal diet is shown in Supplementary Table S1, with composition and nutrient levels in line with the National Research Council (NRC) (1994) in mash form. The trace elements content of the basal diet in control group was based on nutrient requirement for poultry [22], and experimental diets were supplemented with MO on the basis of the control groups’ diet. Sodium molybdate dihydrate (Na_2_MoO_4_•2H_2_O) was used as MO source in this experiment. All hens had *ad libitum* access to experimental diet and water through the whole experiment. Artificial light by a daily lighting schedule of 16 h light and 8 h dark and the temperature were maintained at approximately 22 °C. Hen-day egg production, egg weight, and average feed intake were recorded daily for each cage and the egg production percentage was expressed on the basis of a hen-day. Feed conversion ratio (FCR) was also calculated. At week 12, a total of 20 eggs for each group (4 eggs/replicate) were collected, then egg yolk and egg white were carefully separated and freeze-dried to measure trace elements concentration. 

On the morning of the last day of week 12, the body weight (BW) of all hens was measured, and the 5 hens closest to the average BW and egg production rate from each treatment group were selected. Blood samples were collected from the wing after 12 h of fasting. Serum samples were obtained from these blood samples by incubation at 4 °C for 30 min and subsequent centrifugation at 1500× *g* for 20 min. The same hens were then sacrificed with an overdose intravenous injection of sodium pentobarbital, the liver and kidney were immediately removed. The cecum contents were carefully collected, immediately placed in cryogenic vials, stored immediately at −20 °C in a portable freezer, delivered to the laboratory, and stored at −80 °C until DNA extraction. Sample (1 g) in 10 mL reduced phosphate buffered saline (PBS, 0.1 M, pH 7.2), the cecum material was then suspended by vortexing, and 0.2 mL of the suspension introduced by gavage, into each germfree recipient.

### 2.4. Determination of Metabolic Parameters and Antioxidant Enzyme Activity in Serum

The activities of the aspartate aminotransferase (AST) and alanine transaminase (ALT) were determined using a BS420 Automatic Biochemical Analyzer (Shenzhen Mindray Bio-Medical Electronics Co., Ltd., Shenzhen, China) in accordance with manufacturer’s instructions. 

The activities of enzymes alkaline phosphatase (AKP), lactic dehydrogenase (LDH), superoxide dismutase (SOD), glutathione s-transferase (GSH-ST), glutathione peroxidase (GSH-Px), and xanthine oxidase (XOD) and the levels of total antioxidant capacity (T-AOC) were determined by colorimetric enzymatic assays, and serum malonaldehyde (MDA) concentration was measured by chemical colorimetric method, using an Enzyme-linked Immunosorbent Assay (ELISA) microplate reader (Tecan Co., Grodingen, Austria) and assay kits (AKP, A059-3-1; LDH, A020-1-2; SOD, A001-1-2; GSH-ST, A004-1-1; GSH-PX, A005-1--2; XOD, A002-1-1; T-AOC, A015-1-2; MDA, A003-1), which were purchased from Nanjing Jiancheng Bioengineering Institute of China. All assays were conducted and interpreted according to the manufacturer’s manual without any modification.

### 2.5. Determination of Trace Element in Serum, Egg, and Tissues

The serum and tissue samples and internal standard solution (containing Germanium for Se, Rhodium for Cu and Zn) were vortex-mixed for 10 s before inductively coupled plasma-mass spectrometry (ICP-MS) analysis. The serum concentrations of MO, Cu, Fe, Zn, and Se were measured with a quadrupole ICP-MS (7900x ICP-MS system, Agilent Technologies, Santa Clara, CA, USA) as previously described [23].

### 2.6. DNA Extraction and Intestinal Microbiota Analysis

Microbial DNA was extracted from cecum contents using the QIAamp DNA Stool Mini Kit (QIAGEN, CA, Hamburg, Germany) according to the manufacturer’s instructions. Total DNA was eluted in 50 μL of elution buffer and stored at −80 °C until measurement in the PCR by LC-Bio Technology (Hangzhou, China), and the isolation was confirmed by 1.2% agarose gel electrophoresis. Before sequencing, the above 16S rDNA V3–V4 region of each sample was amplified with a set of primers targeting the 16S rRNA gene region. Sequencing libraries were generated using NEB Next Ultra DNA Library Prep Kit for Illumina (New England Biolabs, Ipswich, MA, USA) following manufacturer’s recommendations and index codes were added. The library quality was assessed on the Qubit@ 2.0 Fluorometer (Life Technologies, Carlsbad, CA, USA) and Agilent Bioanalyzer 2100 system. At last, the library was sequenced on an Illumina MiSeq platform and 300 bp paired-end reads were generated. Metagenomic sequencing was conducted using HiSeq4000 and PE150 strategy. The integrity of the extracted genomic DNA was assessed by electrophoresis on a 1% (w/v) agarose gel. Sequencing and bioinformatics analyses were performed by Novogene Bioinformatics Technology Co. (Tianjin, China). Richness and diversity estimations used the α diversity index including Shannon, Chao1, ACE, and Simpson. Linear discrimination analysis coupled with effect size (LEfSe) analysis used the Kruskal–Wallis rank-sum test with a normalized relative abundance matrix to detect features with significantly different abundances between assigned taxa and performs LDA to estimate the effect size of each feature. Linear discrimination analysis coupled with effect size (LEfSe) was performed to analyze the bacterial taxa differentially represented between the 4 treatments at different taxonomy levels.

### 2.7. Statistical Analysis

All data (except microbiota data) were analyzed by two-way ANOVA using GLM procedure of SAS 9.2 (SAS Institute, Cary, NC, USA) and GraphPad Prism 6.0 (GraphPad Inc., La Jolla, CA, USA). The final model included the main effects of MO and TP and their interaction. The results are presented as mean ± SD. When significant (*p* ≤ 0.05) interactions were observed, the means were compared based on the Tukey’s test. 

## 3. Results

### 3.1. Production Performances

Egg production was decreased by high MO levels in the diet (*p* = 0.03), while dietary TP reversed this (*p* < 0.01; Supplementary Table S2). Egg yolk color was lower in layers fed 100 mg/kg MO (*p* < 0.01), however, TP supplementation was not able to reverse this effect (*p* > 0.05; Supplementary Table S3). No differences among treatments were observed for egg weight, average daily feed intake (ADFI), FCR, eggshell quality, albumen height, Haugh unit (HU), and weight of eggshell, yolk, and albumen (*p* > 0.05).

### 3.2. Trace Element Content in Tissues, Egg, and Serum

**Figure 1:**
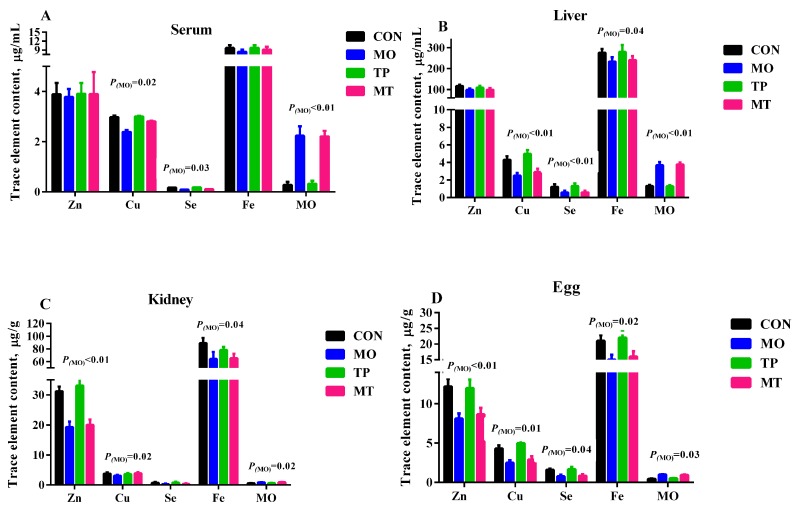
Effect of molybdenum and tea polyphenols on the trace element content in serum and tissues. (**A**) Trace element content in serum; (**B**) Trace element content in liver, (**C**) Trace element content in kidney; (**D**) Trace element content in egg (dry matter basis).

As expected, feeding high MO levels in layer’s diet resulted in higher MO levels in serum, liver, kidney, and egg, and lower Cu and Se content in serum, liver, and egg (*p* < 0.05; Figure 1). Iron concentration in liver, kidney, and eggs was significantly lower in MO supplementation groups (*p* < 0.05). Dietary TP did not affect serum concentration of trace elements measured in this study (*p* > 0.05).

### 3.3. Serum Antioxidant Status Parameters

As shown in Supplementary Table S4 and Table 1, high MO levels in layers’ diet increased ALT, AST, and MDA serum concentration, while it decreased the T-AOC and GSH concentration and the GSH-ST, XOD activity (*p* < 0.05). Dietary TP was able to reverse the effect of MO in ALT, AST, and MDA (*p* < 0.05). 

### 3.4. Microbiota Composition in the Layer Cecum

**Figure 2:**
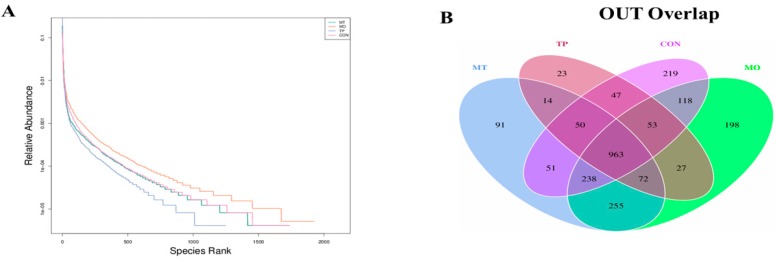

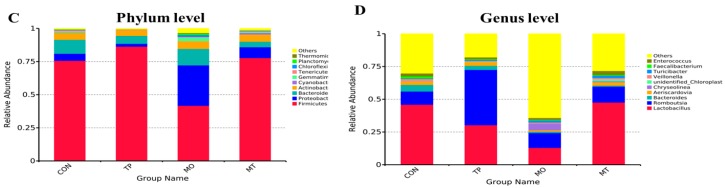
Rank abundance curve of bacterial OTUs derived from each sample (**A**). Venn diagram illustrated in cecum microbiota among the samples (**B**). The relative abundance of the top 10 phylum from samples (**C**). Bar graph of the top 10 genus from samples (**D**).

Relative microbial abundances of the cecum at phylum level indicated that *Firmicutes* was the dominant phylum in all dietary treatments (CON 75.68%, MO 41.77%, TP 86.36%, and MO + TP 77.78%; Figure 2). *Firmicutes, Proteobacteria,* and *Bacteroidetes* comprised 91.54%, 84.64%, 94.49%, and 90.08% of the microflora in the CON, MO, TP, and MO + TP groups, respectively (Table 2). The MO group had lower *Firmicutes* and higher *Proteobacteria* abundance (*p* < 0.01), while both the TP and MO + TP groups presented in increased abundance in *Firmicutes* abundance (*p* < 0.05) and *Firmicutes/Bacteroidetes* ratio (*p* < 0.05) at phylum level. At the genus level, we observed that the abundance of *Lactobacillus* was increased in the MO + TP group (*p* = 0.01), while the abundance of *Romboutsia* was decreased in the MO group (*p* = 0.02) and increased by dietary TP (*p* = 0.01); this increase was more pronounced in the TP + MO group (*p* = 0.01). The shared and specific OTUs among 4 groups are shown in Figure 2. These data showed that while MO and TP caused microbial variations but did not change the dominant species at phylum level in the cecum.

### 3.5. Alpha Diversity of Gut Microbiota in the Cecum-Fed High MO and TP

To analyze the microbiota composition in the cecum in the 4 treatment groups, OTUs with a cut-off of 97% similarity were identified (Table 3). The indices of Shannon, Chao1, and Simpson were calculated to evaluate the alpha diversities of the microbiota in the CON, MO, TP, and MO + TP groups. The OTUs and Shannon index was increased by high levels of MO (*p* ≤ 0.05) but decreased by dietary TP (*p* < 0.05). 

### 3.6. Beta Diversity of Gut Microbiota in the Cecum Fed High MO and TP

**Figure 3:**
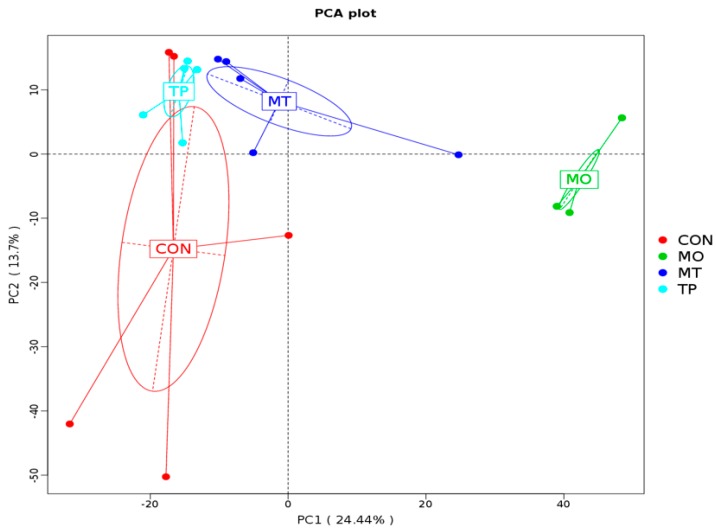
Principal coordinate analysis plot of the cecum microbiota based on the unweighted UniFrac metric.

**Figure 4:**
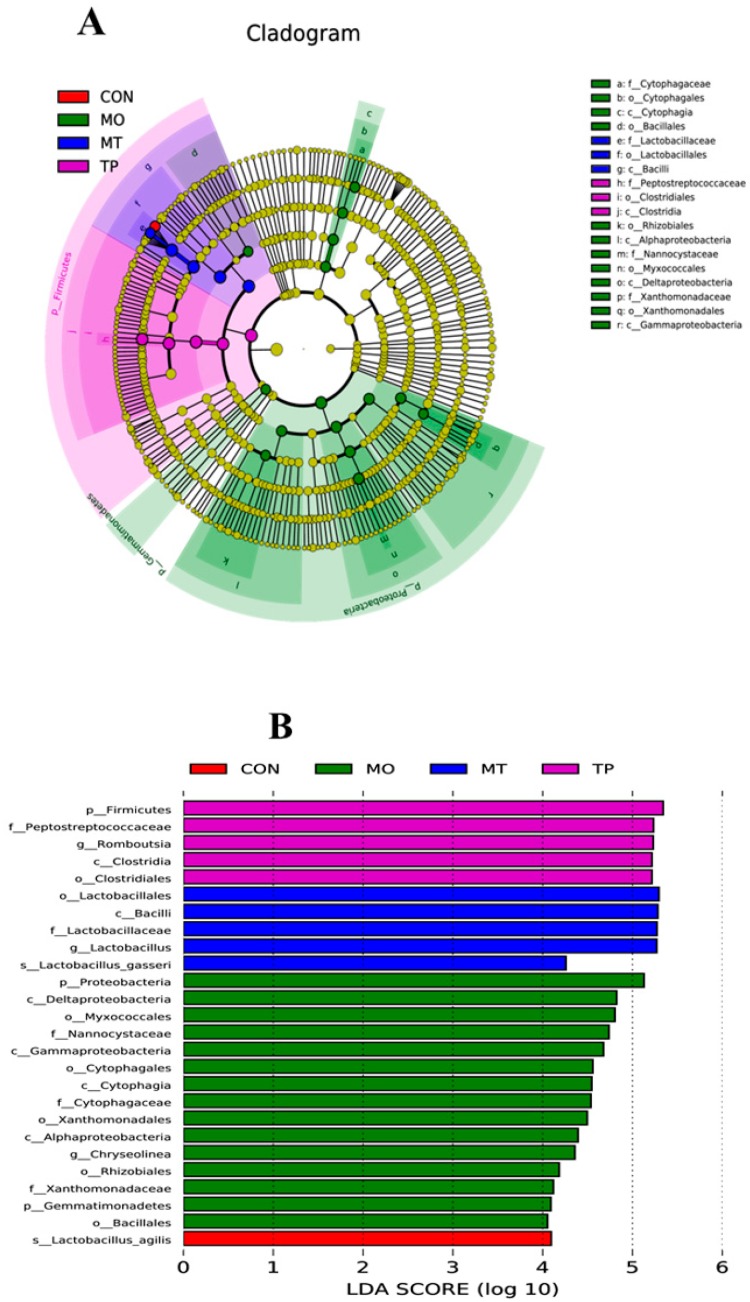
Linear discrimination analysis coupled with effect size (LEfSe) identified the most differentially abundant taxa in the cecum microbiota of MO-fed layers. (**A**) Taxonomic cladogram obtained from LEfSe analysis of 16SrRNA sequencing. Biomarker taxa are heighted by colored circles and shaded areas. Each circle’s diameter is relative to abundance of taxa in the community. (**B**) Only taxa meeting an LDA significant threshold > 3 are shown. (Red) CON enriched taxa; (Green) MO enriched taxa; (Blue) MT enriched taxa; (Purple) TP enriched taxa.

The results indicated that the microbiota of cecal samples was clearly differentiated among CON, MO, and MO + TP groups, whereas the separation between CON and TP could be hardly detected (Figure 3). As shown in Figure 4, MO increased the abundance of many microbial taxa such as *Proteobacteria* (phylum), *Deltaproteobacteria* (class), *Mytococcales* (order), *Nanocystaceae* (family), etc., whereas the TP promoted the enrichment of *Lactobacillus agilis* (species). *Bacilli* (class), *Lactobacillates* (order), *Lactobacillus* (family), and *Lactobacillus gasseri* (species) were enriched in the MO + TP group.

## 4. Discussion

Our study indicates that MO negatively affected productive performances, redox balance status, and cecum microbiota of laying hens, while tea polyphenol (TP) mitigated the negative impact of high MO exposure. Trace elements can be absorbed by organisms and bio-accumulated through every step of the food chain up to animals and humans; however, there is an increasing concern about the adverse effects of MO in farm animals. In our current study, we found that high levels of MO in the diet of laying hens decreased egg production rate and egg yolk color. The literature about MO exposure in laying hens is limited, but previous studies conducted in human and rats indicated that high MO can lead to growth depression, impairment of reproductive performance, and renal failure [24,25,26]. Moreover, it has been indicated that exposure to high levels of MO can result in spleen and kidney toxicity in broilers and ducks [10,27,28,29], which may cause growth retardation and reduction in productive performance. In case of liver damage, blood concentration of ALT and AST, the main aminotransferases involved in the synthesis process of nonessential amino acid, would increase, reflecting impaired hepatic ALT and AST activities [30]. It has been reported that excessive MO may result in morphological and functional changes in the liver, kidney, and spleen [10,27]. Xanthophylls, a subclass of carotenoids, are responsible for egg yolk pigmentation [31]. Egg yolk coloration has been shown to be affected by several components within the diet, such as type and concentration of carotenoids [32,33], dietary fat profile, illness, heavy metal, and mycotoxin [17,34]. Many carotenoids have antioxidants properties, which can be utilized to quench excessive free radical formation during oxidative stress and disease [35]. Therefore, it could be argued that high MO disrupted redox balance, resulting in oxidative stress which was responsible for the observed reduced pigmentation in egg yolk. The observed increase in MDA and decrease in antioxidant enzymes in MO-challenged layers are in support of this hypothesis.

Molybdenum is a transition metal that is usually found in the body in either the MO^4+^ or MO^6+^ valence state bound to sulfur or oxygen. As a transition element that easily changes oxidation state, it functions as an electron transfer agent in oxidation–reduction reaction. Molybdenum is a critical component of several enzymes, including sulfite oxidase, XOD, and aldehyde oxidase [36]. High dietary MO levels decreased serum XOD activity in agreement with the result of Zhang et al. [37]. Moreover, in this study, high dietary MO levels resulted in decreased activities of antioxidant enzymes (T-AOC, XOD, GSH-ST) and GSH concentration while MDA levels were increased. The GSH-ST and SOD are one of the primary enzymatic defense mechanisms that are activated against reactive oxygen species, with both enzymes playing an integral role in free radical modulation [38]. Furthermore, XOD provides a substantial contribution to the maintenance of redox balance by catalyzing the conversion of hypoxanthine to xanthine, uric acid, and superoxide [39]. MDA is an oxidized lipid metabolite and can be used to measure the level of lipid peroxidation [40]. The results of this study indicate that high MO disrupted redox balance in layer hens. The maintenance of redox balance is crucial for effective immunity and health and biomarkers of oxidative stress have been linked to gastrointestinal functionality [41,42]. In a previous study, Rio et al. [43] also found that a high dosage of MO can result in decreased activity of antioxidant enzymes and a decline in the antioxidant capacity of the organism. Studies in rabbits have shown that high MO accumulation generates free radical processes or reactive intermediates, resulting in the alteration of MDA and GSH-Px levels [44,45]. In ducks exposed to high levels of MO, an increase in MDA levels and a decrease in XOD and CAT activities in serum and spleen tissue has also been observed [28,37]. Therefore, the toxic effect of exposure to high MO levels can result in oxidative damage. Moreover, high levels of MO are known to interfere with the absorption and metabolism of selenium (Se), copper (Cu), iron (Fe), and zinc (Zn) [6,46]. It has been demonstrated that high MO competes with Zn in the binding sites of protein sulfhydryl, which led to a Zn deficiency. On the other hand, MO can inhibit Fe transportation and utilization, which causes Fe deficiency [6]. In the current study, our data also indicates that dietary MO disrupted the homeostasis of trace elements in laying hen. Indeed, we observed that high MO decreased Se, Zn, Cu, and Fe concentration in serum, liver, kidney, and eggs. Zinc, Cu, and Se are an essential component of several antioxidant enzymes such as Cu-Zn SOD, GSH-Px, and thioredoxin reductase [47]. This may also be one of the reasons that high MO leads to oxidative stress in this study. On the other hand, it has been observed that the administration of EGCG decreased serum levels of AST and ALT, decreased MDA levels in the liver, and remarkably restored the liver activities of SOD and GSH-Px in high-fat-induced obesity mice model [20], but it did not affect the trace element content in serum, tissues, and egg. Therefore, dietary polyphenols can reverse the negative impact of high levels of MO on redox balance.

In ruminants it has been demonstrated that high levels of dietary MO can interfere with microbial metabolism [4,5]; however, information about the effect of MO on the poultry microbiota is limited. In this study, we observed that exposure to high MO levels resulted in lower *Firmicutes* and higher *Proteobacteria* abundance, whereas in the TP and MO + TP groups, an increase in *Firmicutes* abundance and *Firmicutes/Bacteroidetes* ratio at phylum level was observed. This observation suggests that *Firmicutes* are inhibited to a larger extent by polyphenols and their metabolites, thus tilting the balance in favor of *Bacteroidetes* in the gut [48]. The *Firmicutes/Bacteroidetes* ratio is considered a biomarker of gastrointestinal functionality and can be indicative of eubiosis conditions in the gastrointestinal tract [20]. While *Firmicutes* such as *Lactobacillus* and *Lactococcus spp.* have biotechnological value in fermentation and bacteriocin production, many of the phylum’s best-known representatives are pathogens. In the present study, the decrease in the ratio of *Firmicutes/Bacteroidetes* was observed after the consumption of MO, which indicated the modulatory effect of MO on the taxonomy of intestinal microbiota. This may be also associated with the disturbance of MO in trace element absorption and metabolism. It has been seen that MO decreased the Fe content in tissues and serum in current study as shown above. Iron also plays a vital role in host immunity, oxidative stress, and can interfere with the gut microbiota in both human and animals [49]. It has been reported that low dietary Fe can lead to a decrease in *Firmicutes*, *Lactobacillus*, *Bifidobacterium*, and *Prevotella* [50], but the exact mechanisms associated with this need to be further studied. On the contrary, other studies in obese mice models, have indicated that green tea polyphenol EGCG resulted in an increased abundance of *Bacteroidetes* with concomitant decrease of *Firmicutes* and *Firmicutes/Bacteroidetes* ratio [20,21], which is not in agreement with the observation of our study. *Proteobacteria* is a major phylum of gram-negative bacteria, which include a wide variety of pathogens, such as *Escherichia*, *Salmonella*, *Vibrio*, *Helicobacter*, *Yersinia*, *Le gionellales,* and many other notable genera. In current study, we found that MO increased the abundance of *Proteobacteria,* which may be associated with the reducing effect of MO on the egg laying rate. Additionally, an increase in *Proteobacteria* has been reported in condition of dysbiosis and oxidative stress [51]. It has been reported that TP can also alter the abundance of *Proteobacteria* in human and mice model too [52]. In our previous study, EGCG supplementation promoted growth of beneficial bacteria, such as *Bifidobacterium spp.* and *Lactobacillus/Enterococcus* groups similarly to fructo-oligosaccharides [21]. Moreover, we also noted that TP increased the enrichment of *Bacilli* (class), *Lactobacillates* (order), *Lactobacillus* (family), and *Lactobacillus gasseri* (species) in MO-challenged layers, which suggests that TP can reverse the adverse effect of high levels of MO the cecum microbiota. 

## 5. Conclusions

The results gathered in this study suggest that dietary supplementation with high MO led to reduction in production performance and disrupted redox balance, while the addition of TP in the diet can reverse these effects. Microbiota analysis revealed that MO and TP differentially enriched microbial compositions in the cecum of layer hens; it seems that dietary TP in MO-challenged layers can maintain eubiosis of the cecum microbiota. 

## Figures and Tables

**Table 1:** Effect of molybdenum and tea polyphenols and serum antioxidant status of laying hens.

Items ^1^		T-AOC, U/mgprot	GSH-ST, U/mgprot	GSH, μmol/gprot	SOD, U/mgprot	MDA, mmol/gprot	GSH-Px, U/mgprot
MO, mg/kg	TP, mg/kg						
0	0	5.40 ± 0.46 ^a^	110.61 ± 23.21 ^a^	89.48 ± 16.31 ^b^	20.34 ± 4.10	4.76 ± 1.21 ^b^	1706.1 ± 189.2
0	600	4.80 ± 0.29 ^a^	116.05 ± 16.20 ^a^	106.94 ± 11.32 ^a^	19.42 ± 2.78	5.72 ± 0.78 ^b^	1616.8 ± 98.3
100	0	3.55 ± 0.54 ^b^	69.35 ± 23.22 ^b^	47.72 ± 16.20 ^c^	13.89 ± 5.77	8.57 ± 1.32 ^a^	1469.1 ± 210.1
100	600	3.84 ± 0.33 ^b^	99.95 ± 19.19 ^a,b^	82.20 ± 17.81 ^b^	18.42 ± 8.21	6.45 ± 1.89 ^b^	1942.6 ± 245.8
*p*-Value		0.06	0.03	<0.01	0.28	0.04	0.73
*p*-Value							
MO		0.04	<0.01	0.40	0.41	0.01	0.96
TP		0.16	0.12	0.11	0.43	0.65	0.18
MO*TP ^2^		0.29	0.04	0.01	0.16	0.01	0.53

^1^ Each mean represents 5 cages, with 2 layer/cage. Abbreviations represented: TP = tea polyphenols; MO = molybdenum; T-AOC = total antioxidant capacity; GSH-ST = glutathione s-transferase; GSH = glutathione; SOD = superoxide dismutase; MDA = malonaldehyde; GSH-Px = glutathione peroxidase. ^2^ MO*TP means the interaction between MO and TP. ^a,b^ Means in the same column with different letters differ significantly (*p* < 0.05).

**Table 2:** Effect of molybdenum and tea polyphenols on top 5 phylum abundance of cecum microbiota of laying hens.

Items ^1^		Firmicutes	Proteobacteria	Bacteroidetes	Actinobacteria	Gemmatimonadetes	Firmicutes/Bacteroidetes
MO, mg/kg	TP, mg/kg						
0	0	75.68 ± 6.78 ^a^	5.34 ± 2.18^b^	10.52 ± 4.25	5.25 ± 1.21	0.79 ± 0.09 ^b^	23.10 ± 8.24 ^a^
0	600	86.36 ± 8.99 ^a^	2.15 ± 0.89^b^	5.98 ± 2.34	4.26 ± 0.78	0.10 ± 0.03 ^b^	19.29 ± 6.10 ^a^
100	0	41.77 ± 5.78 ^b^	30.48 ± 2.21^a^	12.39 ± 4.21	5.85 ± 0.45	2.52 ± 0.21 ^a^	4.53±0.37 ^b^
100	600	77.78 ± 7.91 ^a^	8.18 ± 1.21^b^	4.12 ± 1.09	5.43 ± 0.37	0.71 ± 0.10^b^	29.46±8.98 ^a^
*p*-Value		<0.01	<0.01	0.28	0.86	<0.01	0.01
*p*-Value							
MO		<0.01	<0.01	0.99	0.52	<0.01	0.60
TP		<0.01	<0.01	0.06	0.61	<0.01	0.19
MO*TP ^2^		0.03	<0.01	0.57	0.84	0.05	0.05

^1^ Each mean represents 5 cages, with 2 layer/cage. Abbreviations represented: TP = tea polyphenols; MO = molybdenum. ^2^ MO*TP means the interaction between MO and TP. ^a,b^ Means in the same column with different letters differ significantly (*p* < 0.05).

**Table 3:** Effect of molybdenum and tea polyphenols on biodiversity of cecum microbiota of laying hens.

Items ^1^		OTU	Chao1	Shannon	Simpson
MO, mg/kg	TP, mg/kg				
0	0	551.4 ± 109.3	1486.1 ± 254.8	5.56 ± 0.76	0.91 ± 0.07
0	600	452.6 ± 145.5	1108.6 ± 227.6	4.45 ± 0.91	0.81 ± 0.11
100	0	815.5 ± 178.2	1865.7 ± 363.7	7.01 ± 0.77	0.96 ± 0.38
100	600	588.0 ± 139.2	1555.4 ± 290.6	5.34 ± 0.44	0.90 ± 0.06
*p*-Value		0.04	0.19	0.04	0.15
*p*-Value					
MO		0.01	0.09	0.05	0.10
TP		0.01	0.15	0.02	0.48
MO*TP ^2^		0.18	0.88	0.61	0.57

^1^ Each mean represents 5 cages, with 2 layer/cage. Abbreviations represented: TP = tea polyphenols; MO = molybdenum. ^2^ MO*TP means the interaction between MO and TP.

## Supplementary Materials

The following are available online at https://www.mdpi.com/2076-3921/8/10/503/s1, Table S1: Composition and nutrient level of basal diet (as-fed basis), Table S2: Effect of molybdenum and tea polyphenols on production performance of laying hens, Table S3: Effect of molybdenum and tea polyphenols on egg quality of laying hens, Table S4: Effect of molybdenum and tea polyphenols on serum characteristics of laying hens, Table S5: Effect of molybdenum and tea polyphenols on genus abundance of cecum microbiota of laying hens.

## Abbreviation

AKP = alkaline phosphatase, ALT = alanine transaminase, AST = aspartate aminotransferase, BW = body weight, CON = control, Cu = copper, EC = epicatechin, ECG = epicatechin-3-gallate, EGC = epigallocatechin, EGCG = epigallocatechin gallate, FCR = feed conversion ratio, Fe = iron, GSH = glutathione, GSH-ST = glutathione s-transferase, ICP-MS = inductively coupled plasma-mass spectrometry, LDH = lactic dehydrogenase, MDA = malonaldehyde, MO = molybdenum, NRC = National Research Council, SOD = superoxide dismutase, T-AOC = total antioxidant capacity, TP = tea polyphenol, XOD = xanthine oxidase, Zn = zinc.
