# Supplementary material for: Alteration of the Antioxidant Capacity and Gut Microbiota under High Levels of Molybdenum and Green Tea Polyphenols in Laying Hens
Source: Antioxidants (Basel). 2019 Oct 22;8(10):503. doi: 10.3390/antiox8100503 (PMC6826559; doi:10.3390/antiox8100503)
Supplement: Supplementary file 1 [file antioxidants-08-00503-s001.pdf]

Supplementary tables:

Table S1. Composition and nutrient level of basal diet (as-fed basis)

Item, %	Amount	
Corn	64.80	
Soybean oil	0.20	
Soybean meal	24.00	
Calcium carbonate	8.66	
Calcium hydrophosphate	1.16	
NaCl	0.35	
Choline Chloride	0.16	
Vitamin premix[1]	0.03	
Mineral premix[2]	0.50	
Analyzed nutrient level, %		
AME[3], kcal/kg	2680	
Crude protein	15.73	
Calcium	3.61	
Available Phosphorus	0.28	
Lysine	0.69	
Methionine	0.34	
Analyzed trace element level	Feed, µg/g	Water, µg/mL
Cd	ND[4]	ND[4]
Pb	ND[4]	ND[4]
Mo	1.5	0.01
Fe	145	0.19
Cu	56	0.03
Zn	138	0.13
Se	0.9	0.01

[1]proviede per kilogram of diet: vitamin A, 8,000 IU; vitamin D_3, 1,600 IU; vitamin E, 5 mg; vitamin K_3, 2 mg; vitamin B_1, 0.8 mg; vitamin B_2, 2.5 mg; vitamin B_6, 1.5 mg; vitamin B_{12},0.04 mg; folic acid, 0.25 mg; niacin, 20 mg; Ca-pantothenate acid,2.2 mg, and biotin, 0.1 mg.

[2]Provided per kilogram of diet: 60 mg Mn (as MnSO4•H2O); 80 mg Zn (as $ZnSO_4$); 8 mg Cu (as $CuSO_4$•$5H_2O$); 60 mg Fe (as $FeSO_4$•$7H_2O$); 0.3 mg Co ($CoSO_4$•$5H_2O$); 0.35 mg I (as KI), and 0.3 mg Se (as Na_2SeO_3•$5H_2O$)

[3]Calculated by NRC (1994).

[4]ND represented not detected.

Table S2. Effect of molybdenum and tea polyphenols on production performance of laying hens[1]

Item		Egg production, %	Egg weight, g	ADFI, g	FCR
MO, mg/kg	TP, mg/kg				
0	0	89.38±1.21[a]	64.7±0.6	119±2	1.92±0.11
0	600	88.69±1.09[a]	64.4±0.5	118±1	1.96±0.07
100	0	84.35±2.01[b]	64.8±0.7	116±2	2.05±0.23
100	600	87.75±1.66[a]	64.0±0.9	116±1	1.96±0.19
P-Value		0.02	0.30	0.44	0.43
P-Value					
MO		0.03	0.91	0.31	0.57
TP		0.38	0.29	0.47	0.22
MO*TP[2]		<0.01	0.09	0.42	0.66

[1]Each mean represents 5 replicates, with 10 layer/replicate. Abbreviations represented: TP = total polyphenols; MO = molybdenum; ADFI = average daily feed intake; FCR = feed conversion ratio.

[2]MO*TP means the interaction between MO and TP.

[a,b] Means in the same column with different letters differ significantly ($p < 0.05$).

Table S3. Effect of molybdenum and tea polyphenols on egg quality of laying hens[1]

Item[1]		Eggshell thickness, mm^{-2}	Eggshell strength, kg/cm^3	Albumen height, mm	Yolk color	Haugh unit	Yolk weight, %	Eggshell weight, %	Albumen weight,%
MO, mg/kg	TP, mg/kg								
0	0	0.36±0.01	3.79±0.67	7.09±0.37	9.44±0.14	83.20±1.34	28.21±0.45	11.30±0.66	60.71±1.66
0	600	0.35±0.02	3.80±0.54	7.05±0.18	9.43±0.07	82.40±1.78	28.88±0.77	11.23±0.77	60.08±0.78
100	0	0.33±0.02	3.73±0.41	7.29±0.25	9.11±0.09	83.76±1.56	27.83±0.54	11.02±0.44	61.07±0.60
100	600	0.34±0.02	3.66±0.35	7.39±0.33	9.14±0.11	84.59±1.32	28.46±0.66	11.00±0.56	60.58±0.98
P-Value		0.93	0.43	0.56	<0.01	0.68	0.62	0.43	0.14
P-Value									
MO		0.55	0.92	0.57	<0.01	0.42	0.20	0.27	0.32
TP		0.82	0.38	0.32	0.31	0.59	0.76	0.69	0.81
MO*TP[2]		0.97	0.13	0.42	0.36	0.63	0.51	0.41	0.16

[1]Each mean represents 5 replicates, with 10 layer/replicate. Abbreviations represented: TP = tea polyphenols; MO = molybdenum.

[2]MO*TP means the interaction between MO and TP.

[a,b] Means in the same column with different letters differ significantly ($p < 0.05$).

Table S4. Effect of molybdenum and tea polyphenols on serum characteristics of laying hens

Items[1]		AST, U/gprot	ALT, U/gprot	AKP, U/gprot	LDH, U/gprot	XOD, U/gprot
MO, mg/kg	TP, mg/kg					
0	0	226.40±20.34[b]	20.10±2.10[b]	380.50±45.62	450.21±35.88	7.89±0.89
0	600	218.22±18.21[b]	21.32±1.41[b]	400.21±55.32	461.20±29.54	8.01±0.54
100	0	330.60±28.21[a]	30.55±2.78[a]	440.32±61.21	520.77±41.44	4.21±1.32
100	600	234.50±22.34[b]	22.75±1.43[b]	410.22±34.98	465.58±57.88	5.14±0.99
P-Value		<0.01	0.04	0.57	0.65	0.05
P-Value						
MO		0.02	0.04	0.71	0.24	0.02
TP		0.33	0.45	0.67	0.78	0.74
MO*TP[2]		0.02	0.02	0.38	0.69	0.51

[1]Each mean represents 5 cages, with 2 layer/cage. Abbreviations represented: TP = tea polyphenols; MO = molybdenum; TC = total cholesterol; TG = total triglyceride; AST = aspartate aminotransferase; ALT = alanine transaminase; AKP = alkaline phosphatase; LDH = lactic dehydrogenase; XOD = xanthine oxidase.

[2]MO*TP means the interaction between MO and TP.

[a,b] Means in the same column with different letters differ significantly ($p < 0.05$).

Table S5. Effect of molybdenum and tea polyphenols on genus abundance of cecum microbiota of laying hens

Items[1]		Lactobacillus	Romboutsia	Bacteroides	Aeriscardovia	Chryseolinea
MO, mg/kg	TP, mg/kg					
0	0	45.95±8.78[a]	10.07±3.12[b]	5.04±2.65	2.53±0.45	0.25±0.12[b]
0	600	30.29±10.12[a]	42.23±8.55[a]	3.12±1.89	2.78±0.21	0.07±0.03[b]
100	0	12.99±6.57[b]	11.33±3.22[b]	0.94±0.54	1.13±0.67	5.07±1.05[a]
100	600	47.64±12.34[a]	12.07±1.98[b]	0.72±0.33	2.61±1.43	0.86±0.51[b]
P-Value		0.05	<0.01	0.22	0.71	<0.01
P-Value						
MO		0.40	0.02	0.06	0.47	<0.01
TP		0.31	0.01	0.51	0.42	<0.01
MO*TP[2]		0.01	0.01	0.61	0.57	<0.01

[1]Each mean represents 5 cages, with 2 layer/cage. Abbreviations represented: TP = tea polyphenols; MO = molybdenum.

[2]MO*TP means the interaction between MO and TP.

[a,b] Means in the same column with different letters differ significantly ($p < 0.05$).
